# Evaluation of a psycho educative group intervention for family members of patients with severe personality disorder undertaken within a mental health centre in Forlì, Italy

**DOI:** 10.1192/j.eurpsy.2025.1909

**Published:** 2025-08-26

**Authors:** R. Casadio, A. Campana, L. Nervegna, C. Barbara, M. Pacetti

**Affiliations:** 1 DSMDP, CSM Forlì, forlì, Italy

## Abstract

**Introduction:**

Severe personality disorders have a significative impact not only on patients but also among their families, causing emotional distress and relevant burden related to care giving. Psycho-educational intervention targeting family members can improve their understanding of the disorder and their ability to cope with symptoms.

**Objectives:**

Forlì Mental Health Centre multi-professional team undertake this study with the scope to evaluate the efficacy of a nine sessions’ psycho-educational program targeting a group of family members, based on the dialectical behavioral therapy (DBT) principles and skills.

**Methods:**

12 participants completed the program and filled self report questionnaires administered at the first (t0) and ninth (t1) session: Symptom Checklist - 90 (SCL-90) and Zarit Burden Interview (ZBI). They also answered a qualitative format investigating their subjective experience.

**Results:**

Results at baseline (t0) indicate high scores on the SCL-90 subscales such as Somatisation (mean = 1,21; st. dev = 0,91), Depression (mean = 1,08; st. dev = 0,86), Sleep disturbances (mean = 1,39; st. dev = 1,03).

Statistical analysis suggest an increase of SCL-90 subscales, at the end of the program (t1), such as Somatisation (mean = 1,38; st. dev = 0,85), Obsessions and compulsions (mean = 1,19; st. dev = 0,78), Depression (mean = 1,40; st. dev = 0,93), Paranoia (mean = 0,93; st. dev = 0,78), Sleep disturbances (mean = 2,18; st. dev = 1,03). ZBI questionnaire total scores indicate an increase of perceived caregiving burden(mean = 42; st. dev = 24,7). Qualitative indicators were administered at the end of the program showing a general satisfaction for the intervention described as “useful”, “important”, “helpful”, “comforting”. The most liked session was the one about “validation” skill.

**Conclusions:**

Despite the perceived psychological distress and burden’ increase, participants have intended the psycho-educative program as useful and worth it. Results suggest that their learnings may be paired with complementary skills and their generalization to enable a significative reduction of psychological impact of caregiving. Future development will include a follow up session to explore this possibility.

**Disclosure of Interest:**

None Declared

